# Supplementary material for: Ovicidal, larvicidal and pupicidal efficacy of silver nanoparticles synthesized by Bacillus marisflavi against the chosen mosquito species
Source: PLoS One. 2021 Dec 17;16(12):e0260253. doi: 10.1371/journal.pone.0260253 (PMC8682912; doi:10.1371/journal.pone.0260253)
Supplement: S1 Table — (DOCX) [file pone.0260253.s001.docx]

**S1 Table**: **Ovicidal activity of AgNPs synthesized by *Bacillus thuringiensis***

**against the eggs of *Ae. aegypti, Cx. quinquefasciatus and An. stephensi***

| Conc.  (ppm) | % mortality for the eggs of *Ae. aegypti* [M(SD)]* | % mortality for the eggs of *Cx quinquefasciatus* [(M(SD)]* | % mortality for the eggs of *An. stephensi* [(M(SD)]* |
| --- | --- | --- | --- |
| 5 | 2(2.31) | 16(3.82) | 0(0.00) |
| 10 | 8(3.26) | 44(3.82) | 1(2.00) |
| 20 | 16(3.26) | 48(2.00) | 2(2.31) |
| 30 | 21(2.00) | 60(3.26) | 6(2.31) |
| 40 | 24(3.26) | 64(6.92) | 13(6.00) |
| 50 | 34(2.31) | 68(2.00) | 26(6.92) |
| 60 | 60(3.26) | 72(2.31) | 46(6.92) |
| 70 | 62(2.31) | 76(3.82) | 53(5.03) |
| 80 | 97(3.82) | 92(3.26) | 78(4.00) |

* **Mean** (**Standard Deviation)**
