# Supplementary material for: Ovicidal, larvicidal and pupicidal efficacy of silver nanoparticles synthesized by Bacillus marisflavi against the chosen mosquito species
Source: PLoS One. 2021 Dec 17;16(12):e0260253. doi: 10.1371/journal.pone.0260253 (PMC8682912; doi:10.1371/journal.pone.0260253)
Supplement: S2 Table — (DOCX) [file pone.0260253.s002.docx]

**S2 Table : Lethal concentrations, R^2^, Regression equations and χ2 values for Ovicidal activity of AgNPs synthesized by *Bacillus thuringiensis* against *Ae. aegypti, Cx. quinquefasciatus and An. stephensi***

| Mosquito species | LC_50_  (LCL-UCL)^*^ | LC_90_  (LCL-UCL)^*^ | R^2^ | Regression equation | χ2 (df=8) |
| --- | --- | --- | --- | --- | --- |
| *Ae. Aegypti* | 36.81  (34.43-39.11) | 75.38  (71.70-79.65) | 0.930 | y=1.037x+11.83 | 25.98 (8) |
| *Cx. quinquefasciatus* | 27.60  (15.51-36.27) | 79.42  (66.99-102.1) | 0.872 | y=0.771x+28.69 | 26.30 (8) |
| *An. stephensi* | 30.33  (25.38-34.68) | 67.14  (61.61-74.24) | 0.900 | y=1.087x+17.04 | 19.34 (8) |

**Note: LC_50_- lethal concentration that kills 50 % of the exposed larvae; LC_90_- lethal concentration that kills 90% of the exposed larvae; LCL – Lower confidential limit; UCL – Upper confidential limit; * - 95% Confidence interval; χ2- Chi-square; df- Degrees of freedom; Table value at 0.05% - 15.507**
