# Supplementary material for: Ovicidal, larvicidal and pupicidal efficacy of silver nanoparticles synthesized by Bacillus marisflavi against the chosen mosquito species
Source: PLoS One. 2021 Dec 17;16(12):e0260253. doi: 10.1371/journal.pone.0260253 (PMC8682912; doi:10.1371/journal.pone.0260253)
Supplement: S3 Table — (DOCX) [file pone.0260253.s003.docx]

**S3 Table**: **Pupicidal Activity of AgNPs synthesized by *Bacillus thuringiensis***

**against the pupae of *Ae. aegypti, Cx. quinquefasciatus and An. stephensi***

| Conc.  (ppm) | % mortality for the pupae of *Ae.aegypti* [M(SD)]* | % mortality for the pupae of *Cx.quinquefasciatus* [M(SD)]* | % mortality for the pupae of *An.stephensi* [M(SD)]* |
| --- | --- | --- | --- |
| 5 | 22(2.31) | 21(2.00) | 27(2.00) |
| 10 | 40(3.26) | 47(3.82) | 37(3.82) |
| 20 | 48(3.26) | 57(2.00) | 56(3.26) |
| 30 | 71(2.00) | 69(2.00) | 70(5.16) |
| 40 | 87(3.82)) | 80(3.26) | 81(2.00) |
| 50 | 93(3.82) | 87(3.28) | 92(3.26) |
| 60 | 98(2.31) | 99(2.00) | 95(2.00) |
| 70 | 99(2.00) | 100(0.00) | 99(2.00) |
| 80 | 100(0.00) | 100(0.00) | 100(0.00) |

* **Mean** (**Standard Deviation)**
