# Supplementary material for: Ovicidal, larvicidal and pupicidal efficacy of silver nanoparticles synthesized by Bacillus marisflavi against the chosen mosquito species
Source: PLoS One. 2021 Dec 17;16(12):e0260253. doi: 10.1371/journal.pone.0260253 (PMC8682912; doi:10.1371/journal.pone.0260253)
Supplement: S4 Table — (DOCX) [file pone.0260253.s004.docx]

**S4 Table: Lethal concentrations, R^2^, Regression equations and χ2 values for pupicidal activity of AgNPs synthesized by *Bacillus thuringiensis* against the pupae of *Ae. aegypti, Cx. quinquefasciatus and An. stephensi***

| Mosquito species | LC_50_  (LCL-UCL)^*^ | LC_90_  (LCL-UCL)^*^ | R^2^ | Regression equation | χ2 (df=8) |
| --- | --- | --- | --- | --- | --- |
| *Ae. Aegypti* | 18.36  (3.44- 27.73) | 56.77  (48.07-69.54) | 0.877 | y=1.041x+30.88 | 21.37 (8) |
| *Cx. quinquefasciatus* | 16.74  (2.90-25.65) | 57.57  (49.95-69.21) | 0.895 | y=0.979x+33.60 | 24.54 (8) |
| *An. stephensi* | 17.26  (5.00-25.49) | 57.78  (50.22-68.31) | 0.909 | y=0.987x+32.97 | 23.60 (8) |

**Note: LC_50_- lethal concentration that kills 50 % of the exposed larvae; LC_90_- lethal concentration that kills 90% of the exposed larvae; LCL – Lower confidential limit; UCL – Upper confidential limit; * - 95% Confidence interval; χ2- Chi-square; df- Degrees of freedom; Table value at 0.05% - 15.507**
