# Supplementary material for: Ovicidal, larvicidal and pupicidal efficacy of silver nanoparticles synthesized by Bacillus marisflavi against the chosen mosquito species
Source: PLoS One. 2021 Dec 17;16(12):e0260253. doi: 10.1371/journal.pone.0260253 (PMC8682912; doi:10.1371/journal.pone.0260253)
Supplement: S5 Table — (DOCX) [file pone.0260253.s005.docx]

**S5 Table**: **Larvicidal Activity of AgNPs synthesized by *Bacillus thuringiensis***

**against *Ae. aegypti, Cx. quinquefasciatus and An. stephensi***

| Conc.(ppm) | % mortality of the larval instars of *Ae. Aegypti* [M(SD)]* | | % mortality of the larval instars of *Cx. quinquefasciatus* [M(SD)]* | | % mortality of the larval instars of *An. stephensi* [M(SD)]* | |
| --- | --- | --- | --- | --- | --- | --- |
|  | **Third instar** | **Fourth instar** | **Third instar** | **Fourth instar** | **Third instar** | **Fourth instar** |
| 5 | 27(3.82) | 22(5.16) | 26(2.30) | 22(2.30) | 30(2.30) | 23(3.82) |
| 10 | 43(3.82) | 42(4.00) | 48(3.26) | 43(4.00) | 41(5.03) | 34(2.30) |
| 20 | 50(5.16) | 48(3.26) | 59(3.82) | 49(2.30) | 58(2.30) | 45(3.82) |
| 30 | 79(3.82) | 71(3.82) | 70(2.30) | 61(4.61) | 75(2.00) | 59(5.03) |
| 40 | 93(2.00) | 85(3.82) | 83(2.00) | 81(2.30) | 85(3.82) | 77(2.00) |
| 50 | 98(2.30) | 92(3.26) | 89(2.00) | 96(2.30) | 97(3.82) | 90(2.30) |
| 60 | 100(0.00) | 98(2.30) | 100(0.00) | 96(2.30) | 99(2.00) | 98(2.30) |
| 70 | 100(0.00) | 100(0.00) | 100(0.00) | 100(0.00) | 100(0.00) | 100(2.00) |
| 80 | 100(0.00) | 100(0.00) | 100(0.00) | 100(0.00) | 100(0.00) | 100(0.00) |

* **Mean**(**Standard Deviation)**
