# Supplementary material for: Ovicidal, larvicidal and pupicidal efficacy of silver nanoparticles synthesized by Bacillus marisflavi against the chosen mosquito species
Source: PLoS One. 2021 Dec 17;16(12):e0260253. doi: 10.1371/journal.pone.0260253 (PMC8682912; doi:10.1371/journal.pone.0260253)
Supplement: S6 Table — (DOCX) [file pone.0260253.s006.docx]

**S6 Table : Lethal concentrations, R^2^, Regression equations and χ2 values for larvicidal activity of AgNPs *synthesized* by *Bacillus thuringiensis* against *Ae. aegypti, Cx. quinquefasciatus and An. stephensi***

| Larval species | Larval instars | LC_50_  (LCL-UCL)^*^ | LC_90_  (LCL-UCL)^*^ | R^2^ | Regression equation | χ2 (df=8) |
| --- | --- | --- | --- | --- | --- | --- |
| *Ae. aegypti* | 3^rd^ | 13.65  (8.99 – 5.54) | 54.01  (43.73-69.66) | 0.827 | y=0.991x+36.48 | 27.22 (8) |
|  | 4^th^ | 18.21  (4.11 –27.24) | 56.89  (48.52-68.95) | 0.887 | y=1.034x+31.17 | 21.70 (8) |
| *Cx.quinquefasciatus* | 3^rd^ | 13.96  (0.76 – 3.23) | 56.51  (48.52-67.78) | 0.895 | y=0.940x+36.88 | 27.94 (8) |
|  | 4^th^ | 19.48  (7.23 -27.74) | 57.80  (49.94-68.91) | 0.902 | y=1.044x+29.66 | 20.76 (8) |
| *An. stephensi* | 3^rd^ | 13.27  (3.76 –23.41) | 55.07  (46.47-67.33) | 0.877 | y=0.956x+37.31 | 27.87 (8) |
|  | 4^th^ | 22.83  (14.44-29.20) | 59.29  (52.83-67.89) | 0.936 | y=1.097x+24.95 | 16.01 (8) |

**LC_50_- lethal concentration that kills 50 % of the exposed larvae; LC_90_- lethal concentration that kills 90% of the exposed larvae; LCL – Lower confidential limit; UCL – Upper confidential limit; * - 95% Confidence interval; χ2- Chi-square; df- Degrees of freedom; Table value at 0.05% - 15.507**
