# Supplementary material for: Ovicidal, larvicidal and pupicidal efficacy of silver nanoparticles synthesized by Bacillus marisflavi against the chosen mosquito species
Source: PLoS One. 2021 Dec 17;16(12):e0260253. doi: 10.1371/journal.pone.0260253 (PMC8682912; doi:10.1371/journal.pone.0260253)

S1 Fig. Mortality curves for ovicidal activity of AgNPs synthesized by *Bacillus thuringiensis* against *Ae. aegypti, Cx. quinquefasciatus and An. stephensi*

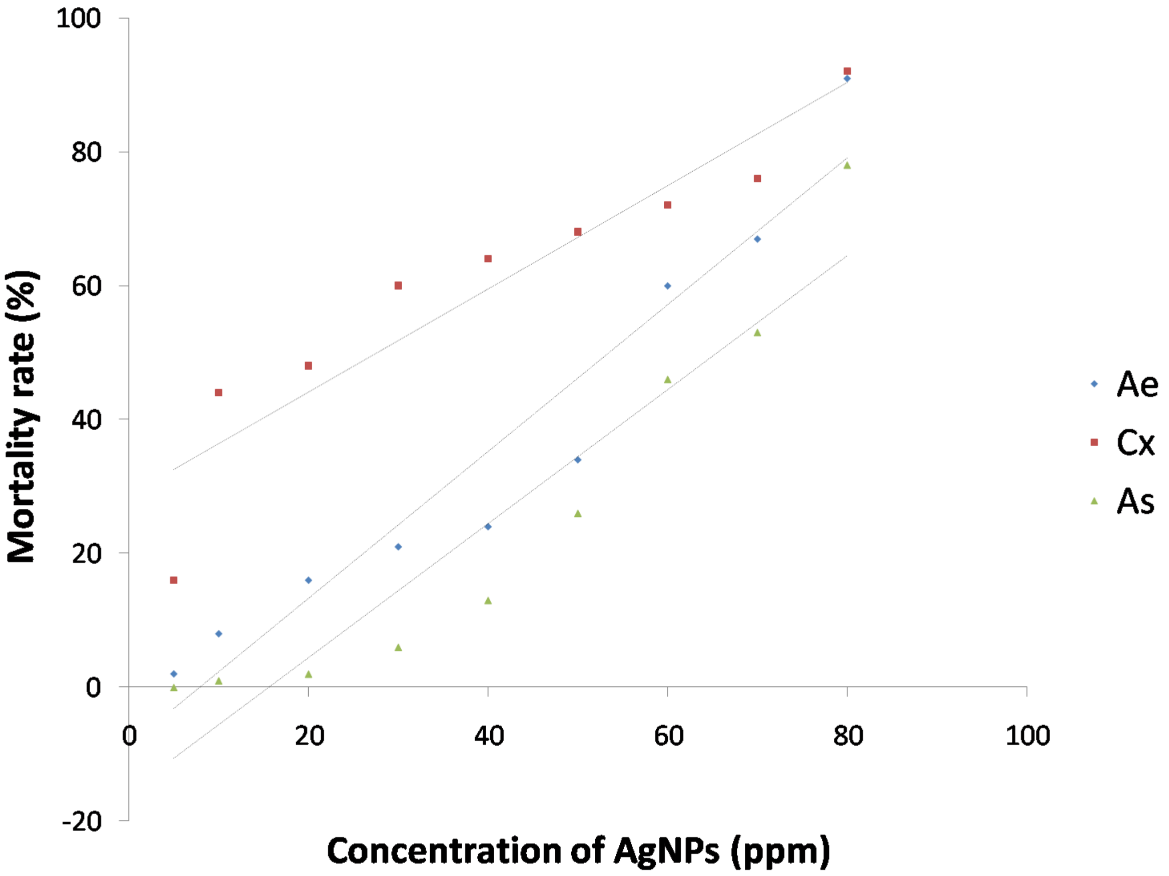

Supplement: S1 Fig — (PDF) [file pone.0260253.s007.pdf]
