# Supplementary material for: Ovicidal, larvicidal and pupicidal efficacy of silver nanoparticles synthesized by Bacillus marisflavi against the chosen mosquito species
Source: PLoS One. 2021 Dec 17;16(12):e0260253. doi: 10.1371/journal.pone.0260253 (PMC8682912; doi:10.1371/journal.pone.0260253)

S2 Fig. Mortality curves for pupicidal activity of AgNPs synthesized by *Bacillus thuringiensis* against the pupae of *Ae. aegypti, Cx. quinquefasciatus* and *An. stephensi*

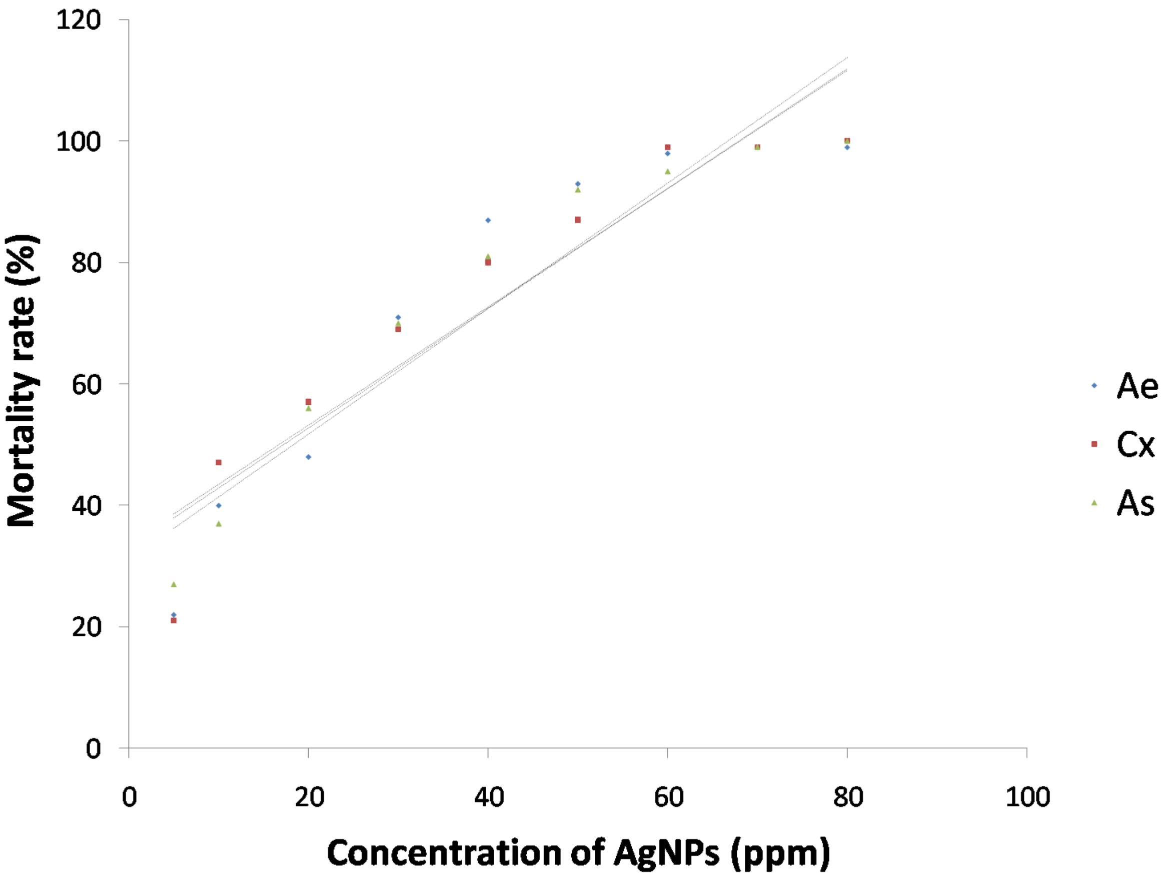

Supplement: S2 Fig — (PDF) [file pone.0260253.s008.pdf]
