# Supplementary material for: Ovicidal, larvicidal and pupicidal efficacy of silver nanoparticles synthesized by Bacillus marisflavi against the chosen mosquito species
Source: PLoS One. 2021 Dec 17;16(12):e0260253. doi: 10.1371/journal.pone.0260253 (PMC8682912; doi:10.1371/journal.pone.0260253)

S4 Fig. Mortality curves for larvicidal activity of AgNPs synthesized by *Bacillus thuringiensis* against 4th instar larvae of *Ae. aegypti, Cx. quinquefasciatus and An. stephensi*

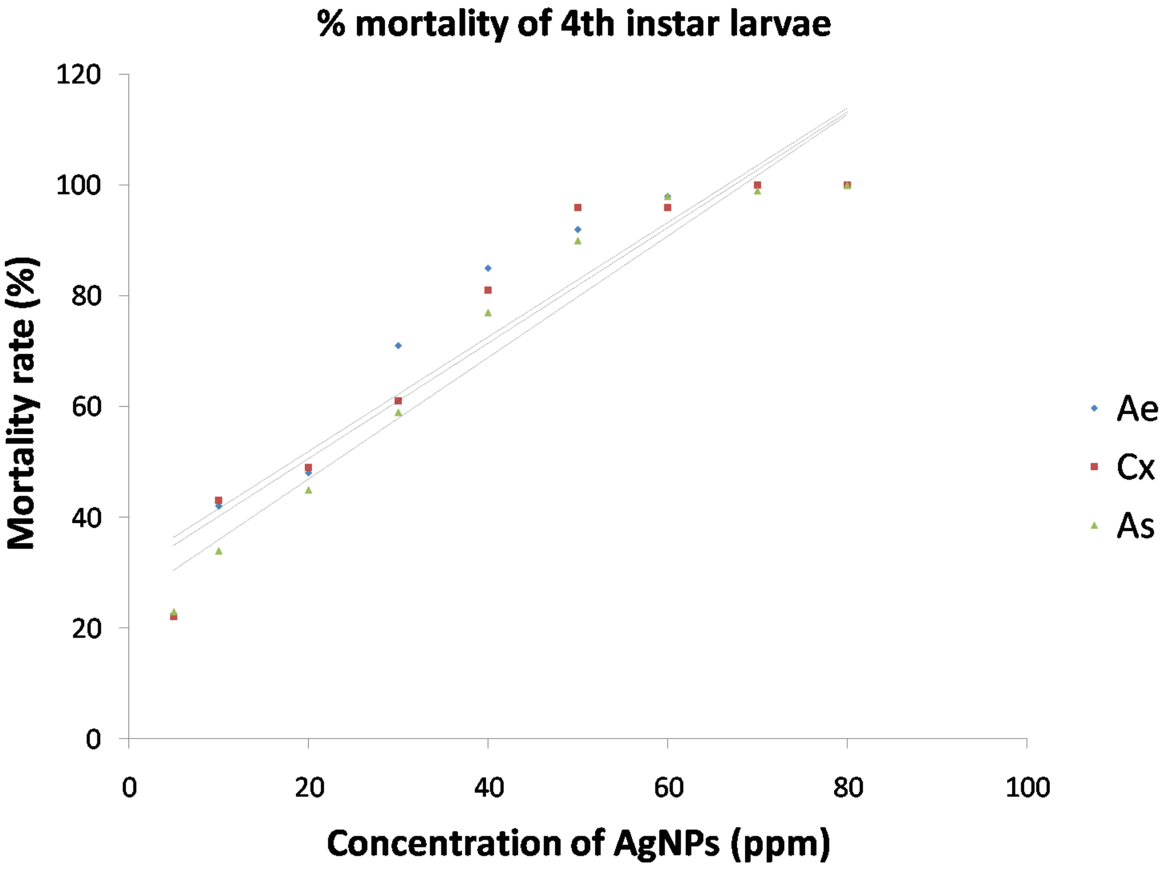

Supplement: S4 Fig — (PDF) [file pone.0260253.s010.pdf]
