# Supplementary material for: Ovicidal, larvicidal and pupicidal efficacy of silver nanoparticles synthesized by Bacillus marisflavi against the chosen mosquito species
Source: PLoS One. 2021 Dec 17;16(12):e0260253. doi: 10.1371/journal.pone.0260253 (PMC8682912; doi:10.1371/journal.pone.0260253)
Supplement: Attachment — Submitted filename: comments on Ms PONE-D-21-03066.docx [file pone.0260253.s011.docx]

**PONE-D-21-03066: Ovicidal, larvicidal and pupicidal efficacy of silver nanoparticles synthesized by Bacillus marisflavi against the chosen mosquito species by Thelma J Fatima College Madurai, Tamilnadu INDIA**

Abstract line 11: disease to humans, better to say disease in humans

Line 20-22 : Sentence not complete: LC50 and LC90 values for the ovicidal, larvicidal and pupicidal efficacy of the AgNPs against the egg, larvae and pupae of Aedes aegypti, Culex 2 quinquefasciatus and Anopheles stephensi respectively.

Line 21-22 : All the observations were statistically 23 significant at P<0.05. Is it all the observations or particular result :

Line 24-26 : Hence, the rapid synthesis of AgNPs would be an appropriate eco-friendly tool for biocontrol of vector mosquitoes.

Line 34-37: Mosquitoes of genus Anopheles (An.) serve as a suitable host for Plasmodium parasite and are 35 responsible for transmitting Malaria. Culex (Cx.) is another genus of mosquito that acts as a host 36 for Wuchereria bancrofti, which transmits Lymphatic filariasis and Aedes (Ae.) that harbors the 37 virus belonging to flaviviridae family and are responsible for causing Dengue.

Line 40: chemical methods; but these

methods aggravate the resistance in mosquitoes,

Line 66-67: Therefore, the present study is intended to explore the nanoparticle synthesizing ability of marine Bacillus species and their efficacy is evaluated against various stages such as egg, larva and pupa of Ae. aegypti, Cx. quinquefasciatus and An. stephensi 69 (L.) respectively under laboratory condition.

Method section is missing

Results and discussion:

Line 73-74 : seawater

Method, results and discussions are mixed

The significance of the regression coefficients can be determined by the P 176 value. P-value is defined as the smallest level of significance leading to rejection of null 177 hypothesis. A small P value indicates a significant study [53]. All the results showed P

Line 175-180: The significance of the regression coefficients can be determined by the P value. P-value is defined as the smallest level of significance leading to rejection of null hypothesis. A small P value indicates a significant study [53]. All the results showed P

Table 2-7: should be presented as probit regression graph (mortality vs concentration) only These tables just present the raw data , further the value of SD should have n value in the parentheses

Materials and methods presented after results and discussion is very confusing and typo errors are seen ,linguistic information is required
