# Supplementary material for: Ovicidal, larvicidal and pupicidal efficacy of silver nanoparticles synthesized by Bacillus marisflavi against the chosen mosquito species
Source: PLoS One. 2021 Dec 17;16(12):e0260253. doi: 10.1371/journal.pone.0260253 (PMC8682912; doi:10.1371/journal.pone.0260253)
Supplement: Attachment — Submitted filename: Response to reviewers.docx [file pone.0260253.s012.docx]

**Rebuttal letter to manuscript**

**PONE-D-21-03066
Ovicidal, larvicidal and pupicidal efficacy of silver nanoparticles synthesized by *Bacillus marisflavi* against the chosen mosquito species
PLOS ONE**

Dear Editor

We would like to thank you for your comments and suggestions, which gave us the opportunity to improve the paper. In the revised manuscript, we have tried to solve all the issues raised. In this document we answer to all the questions raised by the reviewers. Comments are shown in bold font, followed by our answer in normal font. The major corrections in the manuscript are highlighted in yellow.

**Reviewer #1: The study is well performed and the meaning and the logic are clear. The authors prepared the Ag nanoparticles using Bacillus spp is very interesting. However, I am providing some minor suggestions to improve the paper below:**

**Correction needed:

AFM results need some revisions.**

Based on the topographical image given by the AFM analysis, it is interpreted as spherical nanoparticles. It would be helpful, if you indicate what type of result is needed specifically.

**The Conclusion need to be rewritten**

Yes, it is rewritten
 **Noticed, numerous typographical grammatical errors. Authors please revise the whole stuff.**

Yes, all errors corrected
 **Ensure italics of organism’s genus and species name throughout the manuscript.**

Yes, all genus and species italicized
**Check for references as per the journal format.**
Yes, reference style changed
**Line no. 425-27. Please check the reference number 17**
Yes, reference changed **Authors, please add recent references**

Yes, recent references added (45,46,47,48, 49, 52)

**Reviewer #2: The present manuscript explains that the nanoparticles have an excellent toxic effect against the vector mosquitoes. After synthesis of silver nano particle from Bacillus so the toxicity evaluation has been doen against egg, larvae and pupae of disease causing mosquito species. The present study advocates that the rapid synthesis of AgNPs would be an
appropriate eco-friendly tool for biocontrol of vector mosquitoes.
However after careful perusal of the manuscript I have following observations:

Abstract line 11: disease to humans, better to say disease in humans**

Yes, changes made

**Line 20-22 : Sentence not complete: LC50 and LC90 values for the ovicidal, larvicidal and pupicidal efficacy of the AgNPs against the egg, larvae and pupae of Aedes aegypti, Culex quinquefasciatus and Anopheles stephensi respectively.**

Yes, changes made

**Line 21-22 : All the observations were statistically significant at P<0.05. Is it all the observations or particular result?**

Yes, the LC50 and LC90 values recorder for all observations showed a significant result.
 **Line 24-26 : Hence, it is evident that this the rapid synthesis of AgNPs would be an appropriate eco-friendly tool for biocontrol of vector mosquitoes (Is it only vector mosquito or all mosquitoes?)**

Yes, changes made. As we have experimented with only three mosquito species that acts as vector. However it is applicable to all mosquito species.

**Line 34-37: Mosquitoes of genus Anopheles (An.) serve as a suitable host for Plasmodium parasite and are responsible for transmitting Malaria. Culex (Cx.) is another genus of mosquito that acts as a host 36 for Wuchereria bancrofti, which transmits Lymphatic filariasis and Aedes (Ae.) that harbors the 37 virus belonging to flaviviridae family and are responsible for causing Dengue.**

Yes, sentences reframed

**Line 40: chemical methods; but these these methods aggravate the resistance by in mosquitoes,**

Yes, changes made

**Line 66-67: Therefore, the present study is intended to explore the nanoparticle synthesizing ability of marine Bacillus species and their efficacy is evaluated against vectorvarious stages such as egg, larva and pupa of Ae. aegypti, Cx. quinquefasciatus and An. stephensi 69 (L.) respectively under laboratory condition.**

Yes, changes made

**Results and discussion:
Line 73-74 : marine seawater**

Yes, changes made

**Method, results and discussions are mixed**

Yes, revised accordingly
 **Line 175-180: The significance of the regression coefficients can be determined by the P valueP value. P-value is defined as the smallest level of significance leading to rejection of null hypothesisnull hypothesis. A small P value indicates a significant study [53]. All the results showed P**

Sentence deleted
 **Table 2-7: should be presented as probit regression graph (mortality vs concentration) only These tables just present the raw data, further the value of SD should have n value in the parentheses**

Yes, changes made. Probit regression graph given for table 2-13 and SD values are included in paranthesis for table 2,3,6,10,11

**Materials and methods presented after results and discussion is very confusing and typo errors are seen , linguistic information is required**

Yes, order of presentation is changed
