# Supplementary material for: Ovicidal, larvicidal and pupicidal efficacy of silver nanoparticles synthesized by Bacillus marisflavi against the chosen mosquito species
Source: PLoS One. 2021 Dec 17;16(12):e0260253. doi: 10.1371/journal.pone.0260253 (PMC8682912; doi:10.1371/journal.pone.0260253)
Supplement: Attachment — Submitted filename: Response to the reviewers.docx [file pone.0260253.s013.docx]

**Rebuttal letter to manuscript**

**PONE-D-21-03066**

**Ovicidal, larvicidal and pupicidal efficacy of silver nanoparticles synthesized by *Bacillus marisflavi* against the chosen mosquito species**

**PLOS ONE**

Dear Editor

We would like to thank you for your comments and suggestions, which gave us the opportunity to improve the paper. In the revised manuscript, we have tried to solve all the issues raised. In this document we answer to all the questions raised by the reviewers. Comments are shown in bold font, followed by our answer in normal font. The major corrections in the manuscript are highlighted in yellow.

**1. If the authors have adequately addressed your comments raised in a previous round of review and you feel that this manuscript is now acceptable for publication, you may indicate that here to bypass the “Comments to the Author” section, enter your conflict of interest statement in the “Confidential to Editor” section, and submit your "Accept" recommendation.**

**Reviewer #1: All comments have been addressed**

**Reviewer #3: All comments have been addressed**

**2. Is the manuscript technically sound, and do the data support the conclusions?

The manuscript must describe a technically sound piece of scientific research with data that supports the conclusions. Experiments must have been conducted rigorously, with appropriate controls, replication, and sample sizes. The conclusions must be drawn appropriately based on the data presented.**

**Reviewer #1: Yes**

**Reviewer #3: Yes**

**3. Has the statistical analysis been performed appropriately and rigorously?**

**Reviewer #1: Yes**

**Reviewer #3: Yes**

**4. Have the authors made all data underlying the findings in their manuscript fully available?

The** [**PLOS Data policy**](http://www.plosone.org/static/policies.action#sharing) **requires authors to make all data underlying the findings described in their manuscript fully available without restriction, with rare exception (please refer to the Data Availability Statement in the manuscript PDF file). The data should be provided as part of the manuscript or its supporting information, or deposited to a public repository. For example, in addition to summary statistics, the data points behind means, medians and variance measures should be available. If there are restrictions on publicly sharing data—e.g. participant privacy or use of data from a third party—those must be specified.**

**Reviewer #1: Yes**

**Reviewer #3: Yes**

**5. Is the manuscript presented in an intelligible fashion and written in standard English?

PLOS ONE does not copyedit accepted manuscripts, so the language in submitted articles must be clear, correct, and unambiguous. Any typographical or grammatical errors should be corrected at revision, so please note any specific errors here.**

**Reviewer #1: Yes**

**Reviewer #3: Yes**

**6. Review Comments to the Author

Please use the space provided to explain your answers to the questions above. You may also include additional comments for the author, including concerns about dual publication, research ethics, or publication ethics. (Please upload your review as an attachment if it exceeds 20,000 characters)**

**Reviewer #1: The authors clearly addressed to potential reviewer's comment. Now, this manuscript is precise and can be considered for publication in PLOS ONE.**

**Reviewer #3: I do not remember having reviewed this manuscript previously. In any case, I have gone through the manuscript that has already been reviewed by two others. In the R1 version of the manuscript, the authors have carefully taken into account of all the suggestions made by two reviewers. This has improved considerably the readability of this contribution.
I have a few suggestions that the authors may have to consider about the application of the present work to the field conditions. The limitations suggested here may be added to the conclusion unit or the discussion section.

1. While defeating mosquitoes is the ultimate goal in many tropical regions, the application of different methods in achieving this remains largely impractical.

2. The use of silver nanoparticles from Bacillus shown in this work has probably some advantages compared to other methods such as introduction of exotic fish species to eradicate the mosquitoes

3. However, authors may have to provide cost-effective analysis and possible contamination of nanoparticles in the ambient or their effects on other useful microbes.**

Yes, suggested limitations added to the conclusion part

**7. PLOS authors have the option to publish the peer review history of their article (**[**what does this mean?**](https://journals.plos.org/plosone/s/editorial-and-peer-review-process#loc-peer-review-history)**). If published, this will include your full peer review and any attached files.


If you choose “no”, your identity will remain anonymous but your review may still be made public.


Do you want your identity to be public for this peer review? For information about this choice, including consent withdrawal, please see our** [**Privacy Policy**](https://www.plos.org/privacy-policy)**.**

**Reviewer #1: No**

**Reviewer #3: No**
